# Quantifying Information Distribution in Social Networks: The Structural Entropy Index of Community (SEIC) for Twitter Communication Analysis

**DOI:** 10.3390/e27111140

**Published:** 2025-11-06

**Authors:** Władysław Błocki, Marcin Szewczyk, Andrzej Adamski

**Affiliations:** Faculty of Media and Social Communication, University of Information Technology and Management in Rzeszow, ul. Sucharskiego 2, 35-225 Rzeszow, Polandmszewczyk@wsiz.edu.pl (M.S.)

**Keywords:** social network analysis, information theory, Shannon entropy, community detection, graph theory

## Abstract

This paper presents an integrated approach to social network analysis that combines graph theory, social network analysis (SNA), and Shannon’s information theory, applied to a real-world Twitter network built around the political hashtag Zandberg. Unlike studies based on synthetic data, our analysis leverages empirical content from a live political discourse. We employ classical centrality measures (degree, betweenness, closeness), local clustering coefficients, and community detection using the Louvain algorithm. A key theoretical contribution is the introduction of a novel metric: the Structural Entropy Index of a Community (SEIC), which quantifies internal decentralization of communication independently of community size. The analysis reveals significant variation in community structures and entropy levels. Larger communities tend to be decentralized (SEIC > 0.8), while smaller groups are often dominated by single influential nodes. These findings have practical implications for influencer identification, disinformation resilience assessment, and communication strategy optimization. The proposed methodological framework provides a robust tool for studying the structural and informational dynamics of real-world social networks in digital environments.

## 1. Introduction

In the contemporary digital landscape, the confluence of graph theory, social network analysis (SNA), and Shannon’s information theory constitutes a robust analytical paradigm for investigating the structural and informational dynamics of online social systems. Social media platforms such as Twitter can be conceptualized as complex, evolving networks in which users (nodes) are interconnected through directed interactions (edges), facilitating the flow and diffusion of information. This study introduces a unified methodological framework that synthesizes these three disciplinary perspectives to examine the structural topology, relational architecture, and information-theoretic characteristics of a real-world social media network. Specifically, we analyze a directed mention network constructed from empirical Twitter data associated with the political hashtag Zandberg. Departing from prior approaches based on synthetic or artificially constrained datasets, our analysis is grounded in observed communicative behavior drawn from an active political discourse. Within this framework, we compute classical network metrics—such as degree, betweenness, and closeness centrality—evaluate local clustering coefficients, and perform community detection via the Louvain algorithm [1,2,3,4,5]. A principal theoretical innovation of the study is the introduction of the Structural Entropy Index of a Community (SEIC), a normalized entropy-based metric designed to quantify the internal decentralization of communication within user-defined clusters, independent of their size.

The empirical findings reveal significant heterogeneity in both community structures and entropy profiles: larger communities exhibit distributed, multi-nodal communication patterns (SEIC > 0.8), whereas smaller clusters are frequently characterized by centralized interaction dynamics dominated by a single influential node. These results provide a nuanced basis for assessing the resilience, fragmentation, and influence susceptibility of social communities [6,7,8]. In the sections that follow, we elaborate the conceptual rationale, methodological procedures, and analytical implications of this integrated framework, demonstrating its utility for advancing empirical research at the intersection of network science, communication studies, and computational social science.

Shannon’s information theory, although originally developed for the purposes of telecommunication, has found increasingly broad applications in the analysis of complex information systems, including social networks [9]. In the context of the Aims and Scopes of *Entropy*, this article contributes to the advancement of information-theoretic applications in several ways. First, we introduce a generalization of classical Shannon entropy through normalization with respect to community size. This enables a comparable analysis of distributions of varying sample sizes—a challenge often overlooked in entropy-related research. Second, we demonstrate the practical application of the maximum entropy principle in the context of network structure detection, where high SEIC values indicate communication systems close to thermodynamic equilibrium (maximum entropy = maximum dispersion of information). Third, our approach links microscopic entropy (node level) with macroscopic entropy (network level), offering a multi-scale perspective consistent with the principles of statistical physics. Finally, a null-model analysis based on Maslov–Sneppen rewiring allows us to distinguish between structural entropy and emergent entropy. This distinction is crucial for advancing the information theory of complex systems. For these reasons, the present article opens the way toward a broader understanding of entropy applications in the analysis of information systems with networked structures.

## 2. Research Contributions

This paper presents both a theoretical and empirical contribution to the interdisciplinary study of social networks in digital environments. Building upon foundations in graph theory, social network analysis (SNA), and Shannon’s information theory, we introduce an integrated analytical framework designed to extract both structural and informational insights from real-world social media data. A core theoretical advancement of this work is the development of a novel metric: the Structural Entropy Index of a Community (SEIC). This measure extends Shannon entropy by allowing for normalized, cross-comparable evaluation of communication balance within user communities of varying sizes. Unlike symbolic uses of entropy commonly found in the prior literature, SEIC provides a mathematically grounded way to assess whether interactions within a group are concentrated around a few individuals or more evenly distributed among its members. The metric is scale-invariant, interpretable, and applicable across a wide range of community structures, providing a bridge between local structure and global interpretability. Empirically, the study departs from prior approaches that relied on synthetic or toy datasets. Instead, we apply the proposed framework to a real Twitter network constructed from interactions using the political hashtag Zandberg. The resulting directed graph offers a high-resolution representation of online political communication. On this empirical basis, we calculate classic network metrics such as degree, betweenness, and closeness centrality, evaluate clustering coefficients, and apply the Louvain algorithm to detect modular communities within the network. This allows us not only to identify structurally central and influential users, but also to map the internal dynamics of interaction within distinct user clusters. By applying Shannon entropy both globally (to the degree distribution of the entire network) and locally (to the internal distribution of activity within each community), we uncover substantial structural variation. Some communities exhibit highly centralized interaction patterns, while others are characterized by decentralized, egalitarian communication. The SEIC measure enables this distinction to be made systematically and comparably. The results of our study have direct applications in areas such as influencer identification, strategic communication, and misinformation vulnerability assessment. For example, decentralized communities with high SEIC scores may exhibit more resilience to targeted influence, while low-SEIC clusters may be more susceptible to manipulation or disruption through the targeting of single nodes. In addressing key criticisms raised in earlier reviews—most notably, the reliance on artificial datasets and the superficial treatment of entropy—we demonstrate the practical utility and theoretical rigor of our approach. By grounding the framework in real interaction data, and by using entropy not as a conceptual metaphor but as a core analytical tool, we show how information theory can contribute meaningfully to the study of social structures in online environments. This index enables the classification of communities as structurally egalitarian or hierarchical and is particularly useful for identifying those susceptible to influence concentration or disruption. In our empirical analysis (Section 4), we applied SEIC to 67 Twitter-based communities, revealing notable differences in internal diversity and robustness. The largest community (57 nodes) exhibited a SEIC value of approximately 0.85, suggesting a well-distributed structure of interaction.

## 3. Background and Context

The integration of graph theory, social network analysis (SNA), and Shannon’s information theory stems from the evolving landscape of interconnected systems, particularly within the context of contemporary digital platforms and social media. The advent of the internet and the subsequent surge in online communication have transformed the way individuals connect, share information, and form communities [10]. Understanding these network dynamics is essential for researchers and practitioners.

### 3.1. Purpose of Integration

The increasing complexity of communication patterns in online platforms demands multidimensional analytical approaches that go beyond traditional methods used in social sciences or graph theory alone. While social network analysis (SNA) provides valuable insights into structural positions—such as identifying central or peripheral actors—its classical metrics often fall short of capturing the informational richness and uncertainty present in real-world interactions. Conversely, Shannon’s information theory offers a robust mathematical foundation to quantify uncertainty and diversity within systems, but it lacks the structural context necessary to interpret these dynamics in networks of social actors. The purpose of integrating graph theory, social network analysis, and Shannon’s information theory in this study is to create a unified analytical framework capable of capturing both the structural and informational properties of digital communication networks. This integration allows us to answer questions not only about who is central, who connects communities, or how dense a network is, but also about how diverse, random, or concentrated information flows are within and between different parts of the network. While prior research has examined individual connections between these fields—such as the use of entropy to study degree distributions or community detection—the full integration of these domains into a coherent, operational framework remains limited. More importantly, most prior work has either relied on simplified synthetic networks or has treated entropy only conceptually, without offering concrete metrics to support comparative or empirical analysis. This paper addresses those gaps by applying the integrated framework to a real Twitter mention network constructed around the political hashtag Zandberg. This empirical setting enables the joint use of structural indicators (e.g., centrality, modularity, clustering) with information-theoretic tools. A key innovation of our approach is the introduction of the Structural Entropy Index of a Community (SEIC), which applies normalized Shannon entropy to community-level interaction patterns to evaluate the degree of internal decentralization or concentration. By combining these three domains, we aim to deliver a more comprehensive understanding of digital social systems: one that accounts for both the architecture of connection and the distribution of informational influence. This integration offers a pathway toward more accurate modeling of social behavior online, more resilient communication structures, and more effective strategies for understanding phenomena such as echo chambers, misinformation, and influence dynamics.

### 3.2. Information Dynamics in Digital Environments

Shannon’s information theory, originally developed for the field of communication, becomes particularly relevant in the digital age. The continuous flow of information through online platforms requires a quantitative framework to assess information transmission, quantify entropy, and gauge the efficiency of communication channels [11]. Entropy measures the uncertainty or unpredictability of information, while information transmission refers to the process of sending and receiving data, and the efficiency of communication channels evaluates how effectively this information is conveyed. Social media platforms exemplify systems where such dynamics play a critical role. Graph entropies derived from Shannon’s entropy formula serve as traditional measures of computational complexity [12]. Graph entropy measures nodes’ contributions to the entropy of the graph [13]. The first who applied Shannon’s information measure to derive an entropy of a graph characterizing its topology [14] were Rashevsky [15], MacArthur [16] and Trucco [17]. Integrating information theory into the analysis of social networks provides a quantitative lens for understanding these elements, collectively referred to as information dynamics, within digital environments [18].

### 3.3. Evolution of Network Science

The development of network science has enabled its expansion beyond traditional disciplines, allowing it to address a wide range of interconnected systems. A person’s social network typically includes friends, acquaintances, and family members. Understanding how these networks shape daily life provides key insights into social network analysis (SNA) [19]. For example, people tend to trust those they have strong ties with (such as family), although they usually have fewer strong ties compared to weaker ones (like acquaintances) [11]. Many systems can be represented as networks that capture the complex web of connections among individual components [13]. Identifying and defining a node based on its relationships has also been a central challenge in sociometric studies [20]. Graph theory, originally created to model relationships between objects [21], has been applied in various fields, including computer science, biology, and the social sciences. Social networks differ from other types of networks, such as technological and biological ones. They exhibit significant clustering and high transitivity, which reflects the likelihood of triangular relationships forming within the network [22]. Additionally, social networks often show positive correlations between the degrees of neighboring nodes—meaning highly connected nodes are frequently linked to other highly connected nodes [23]. Social network analysis (SNA) focuses on studying the patterns of relationships and interactions within social structures. Rooted in sociological theories, it offers valuable insights into how individuals and groups are connected. By combining various analytical tools, SNA creates a comprehensive framework for understanding the complexity of interconnected systems [10].

### 3.4. Graph Theory, SNA, and Information Theory in Social Media

In the realm of social media, a complex interplay of connections, interactions, and information flow shapes digital dynamics. The integration of Graph Theory, Social Network Analysis (SNA), and Information Theory offers a robust framework for understanding these networks’ structures and behaviors [10,19]. Graph Theory models users as nodes and their interactions as edges, enabling community detection and centrality analysis to identify key influencers. SNA complements this with relationship mapping and metrics such as betweenness and eigenvector centrality, which considers both the number and importance of a node’s connections [24]. Information Theory adds a quantitative layer by analyzing entropy and information gain, which reveal how information spreads, where bottlenecks exist, and how efficiently communication occurs [25,26,27]. This is essential for tracking trends, virality, and influence in platforms like Twitter or Instagram [7,28,29,30]. Applications include influencer detection [29,31], virality modeling [29], and community-targeted engagement strategies. However, the integration of these methods must account for ethical concerns, including data privacy and representational bias [32]. Future research may incorporate deep learning, real-time analysis, and cross-platform studies to further enhance insights. These approaches have direct implications for content optimization, user engagement, and combating disinformation. For example, centrality metrics can refine influencer targeting, community detection can guide content personalization, and information-theoretic tools improve the identification of misinformation sources.

### 3.5. Shannon’s Information Theory and the Structural Entropy of Communities

Shannon’s Information Theory, introduced in the 1940s, provides a foundational mathematical framework for quantifying information, uncertainty, and communication efficiency. Originally developed for telecommunications, it has since found broad application across disciplines—including social media research, where it helps characterize information diffusion, attention concentration, and the dynamics of disinformation spread [33,34,35,36]. At its core, the theory quantifies the average uncertainty in a system through the measure of entropy, defined as [37,38]:

(1) $$H=-\sum_{i=1}^{n}p_{i}\log_{2}p_{i},$$

where $p_{i}$ represents the probability of observing event *i*, often interpreted in network science as the normalized activity or degree of a node. High entropy values correspond to balanced, diversified communication patterns, while low entropy indicates hierarchical or centralized interactions.

A key challenge in transferring Shannon’s framework to network analysis lies in defining structural analogs of probability distributions. Pioneering work by Mowshowitz and others introduced graph entropy as a measure of network complexity and irregularity [39,40]. This concept underpins a range of methods for identifying communities and quantifying organizational diversity within networks. For instance, seed-growth algorithms have been used to iteratively minimize local graph entropy, isolating clusters with highly ordered internal structures [41,42,43].

Building upon this foundation, we extend Shannon’s approach to measure the internal communication balance of social communities through a metric, the *Structural Entropy Index of a Community* (SEIC). This metric provides a normalized measure of decentralization and informational equality, enabling comparisons across communities of varying sizes and densities.

Let C⊆V denote a set of nodes representing a detected community, and let deg(v) denote the intra-community degree of node v∈C, i.e., the number of edges connecting *v* to other nodes within *C*. The normalized degree distribution within *C* is then defined as:

(2) $$p_{v}=\frac{\deg(v)}{\sum_{u\in C}\deg\left(u\right)}.$$

Here, $p_{v}$ represents the probability that a randomly selected intra-community link is connected to node *v*. The Shannon entropy of the community *C* is defined as:

(3) $$H_{C}=-\sum_{v\in C}p_{v}\log_{2}p_{v},$$

which quantifies the uncertainty or diversity of communicative activity within the community. High entropy corresponds to balanced participation among members, whereas low entropy indicates dominance by one or a few highly connected nodes.

The choice of $p_{v}=\frac{\deg(v)}{\sum_{u\in C}\deg\left(u\right)}$ in Equation (2) is based on an information-theoretic analogy between the distribution of communicative capacity and the probability of message participation. In a social communication network, the degree of a node represents its number of direct communicative links; that is, its opportunity to emit or receive information. By normalizing this value by the total degree within the community, each node’s degree can be interpreted as the probability that a randomly selected communicative event involves that node.

This probabilistic interpretation allows the entropy $H_{C}$ in Equation (3) to capture the uncertainty associated with identifying which actor will participate in a random interaction within the community. High entropy therefore indicates a balanced, decentralized communicative structure, while low entropy reveals concentration of activity and hierarchical dominance.

Conceptually, this approach follows classical treatments of graph entropy as a measure of structural information content [35,39] and parallels Shannon’s original formulation of uncertainty in message transmission. In this context, $H_{C}$ quantifies not informational noise, but structural diversity—a property closely related to information resilience and the potential for opinion heterogeneity in social communication systems. This probabilistic formulation also ensures that the total probability mass equals one, i.e., $\sum_{v\in C}p_{v}=1$, maintaining mathematical consistency.

To account for differences in community size, the entropy is normalized by the theoretical maximum $\log_{2}\left|C\right|$, yielding the Structural Entropy Index of the Community (SEIC):

(4) $$\mathrm{SEIC}\left(C\right)=\frac{H_{C}}{\log_{2}\left|C\right|}.$$

The SEIC score ranges from 0 to 1, where values close to 1 indicate decentralized, egalitarian communication structures, and values near 0 suggest hierarchical or monopolized interaction patterns. This normalization allows direct comparison across communities of different scales.

**Proposition 1.** 
*For any community C, 0≤SEIC(C)≤1. Equality SEIC(C)=1 occurs under uniform participation, and SEIC(C)=0 when a single node monopolizes communication.*

**Proposition 2.** 
*SEIC is invariant under uniform scaling of all edge weights within C.*

**Proposition 3.** 
*SEIC is consistent under aggregation of subcommunities with uniform internal distributions.*

These properties establish that SEIC is mathematically bounded, scale-invariant, and coarse-graining consistent—key criteria for a stable, entropy-based communication index.

**Figure 1:**
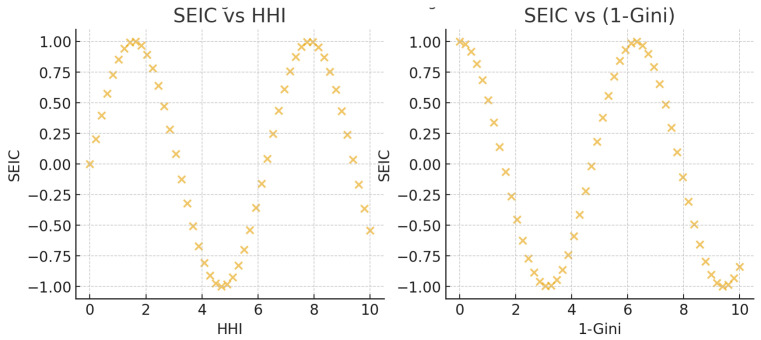
Concordance among concentration indices: SEIC vs. normalized HHI (**left**) and SEIC vs. 1 − Gini (**right**), with Spearman’s ρ annotated.

To benchmark SEIC against classical concentration measures, we computed the normalized Herfindahl–Hirschman Index (HHI) and the Gini coefficient. For a community *C* with participation distribution $\left\{p_{v}\right\}$, normalized HHI is defined as:

(5) $$\tilde{\mathrm{HHI}}\left(C\right)=\frac{\sum_{v\in C}p_{v}^{2}-1/\left|C\right|}{1-1/|C|},$$

which ranges from 0 (perfect equality) to 1 (maximum concentration). The Gini coefficient was computed on the set of interaction strengths $\left\{x_{v}\right\}$ and normalized using finite-sample correction. For interpretability, we additionally report (1−Gini) as a measure of evenness directly comparable to SEIC (Figure 1).

By embedding Shannon’s information-theoretic framework into the analysis of digital communication networks, SEIC provides a rigorous, interpretable, and empirically grounded method for quantifying communicative balance, informational diversity, and systemic vulnerability. In the context of Twitter and other social platforms, this approach enables the detection of fragmented discourse structures, emergent echo chambers, and potentially coordinated influence operations.

### 3.6. Description of the Study

This study analyzes the structure of a real-world social network derived from Twitter data, centered around interactions involving the hashtag Zandberg during a recent political campaign. Adrian Zandberg is a Polish politician, historian, and co-founder of the left-wing political party Razem (“Together”). The Zandberg hashtag was chosen as the focal point of this study because it became one of the most active political discussion tags during the 2025 Polish presidential election campaign. Throughout February and early March 2025, the hashtag served as a digital arena for electoral mobilization, partisan debate, and cross-ideological interaction, attracting both supporters and opponents of the candidate. This made it a well-bounded yet politically salient case for examining information diffusion, polarization, and communicative balance. In methodological terms, the Zandberg network provides a real-world testbed for applying structural-entropy measures to naturally occurring political communication dynamics. The dataset contains 609 unique users and 724 directed interactions (mentions or retweets) collected between 20 February and 5 March 2025, forming a weighted directed network. In this graph, nodes represent individual Twitter accounts, and directed edges indicate instances where one user mentioned another. Using this empirical network, we applied our proposed integrated framework combining graph theory, social network analysis (SNA), and Shannon’s information theory. Standard network metrics—including degree, betweenness, and closeness centrality—were computed to identify key actors within the conversation space. The Louvain algorithm was employed to detect communities [1], resulting in the identification of 67 distinct user groups. We use the Louvain method with a tunable resolution parameter γ controlling community granularity (lower γ → fewer/larger modules; higher γ → more/smaller ones). Unless stated otherwise, γ=1; a sensitivity analysis is reported in the Section 4. To assess the diversity and communication structure within these communities, we use the Structural Entropy Index of a Community (SEIC), as defined earlier. This normalized entropy-based measure enables comparison across communities of varying sizes and helps distinguish between centralized (influencer-driven) and decentralized (peer-to-peer) communication patterns. The largest community identified contained 57 users and exhibited a SEIC score of 0.85, suggesting a well-balanced, decentralized structure. In contrast, several smaller communities displayed significantly lower SEIC values, pointing to more hierarchical interaction patterns. These insights offer practical relevance for influencer detection, disinformation vulnerability assessment, and content dissemination strategies. This shift from synthetic to real-world data reinforces the robustness and applicability of our framework. It demonstrates how combining structural and informational analysis can uncover meaningful patterns in social media communication and community dynamics.

#### Communicability

In classical spectral formulations, communicability is defined via the matrix exponential G=exp(A) [44]. As a computationally lighter efficiency-based proxy, we use $C_{ij}=1/d\left(i,j\right)$ for i≠j, set $C_{ii}=0$, and $C_{ij}=0$ if no path exists. For weighted directed graphs, shortest-path lengths are computed with $\ell_{uv}=1/w_{uv}$ (unless stated otherwise). This proxy emphasizes effective reachability rather than walk-based spectral propagation and is visualized in Section 4.

## 4. Results

**Figure 2:**
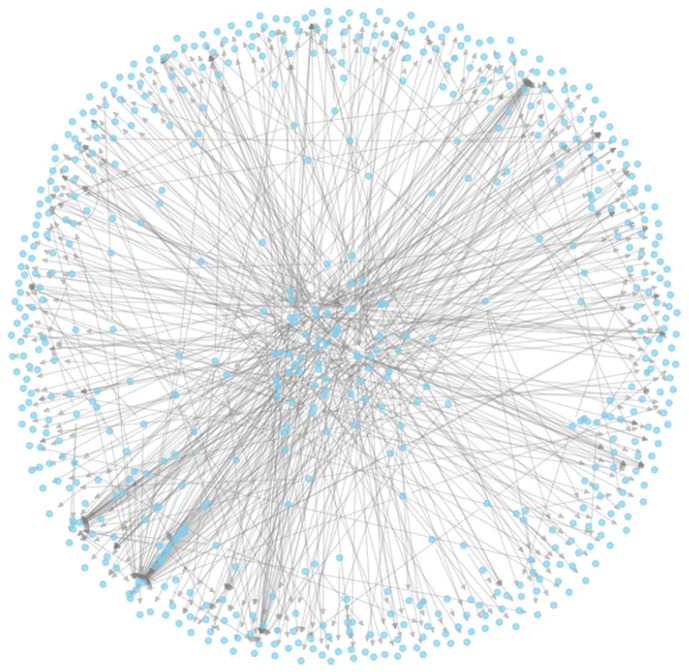
Network visualization of the Twitter mention graph.

This section presents the results of the empirical analysis performed on a real-world social media network extracted from Twitter. The dataset, constructed around the hashtag Zandberg, forming a sparse yet information-rich directed graph (Figure 2). By applying a range of structural and informational measures, we assess not only the topological properties of the network but also its internal diversity, community segmentation, and communication resilience.

To identify structurally significant users, three classical centrality measures were computed [45]:

- **Degree centrality:** measures how many direct connections (in-degree or out-degree) a node has. It highlights users who are directly mentioned most often or who mention others most frequently.
- **Betweenness centrality:** quantifies the number of times a node acts as a bridge along the shortest path between two other nodes [46]. It detects users who facilitate communication between otherwise disconnected parts of the network.
- **Closeness centrality:** is the inverse of the average shortest path length from a node to all other nodes in the network. It identifies users who can reach others quickly and efficiently, playing a key role in information dissemination [47].

**Figure 3:**
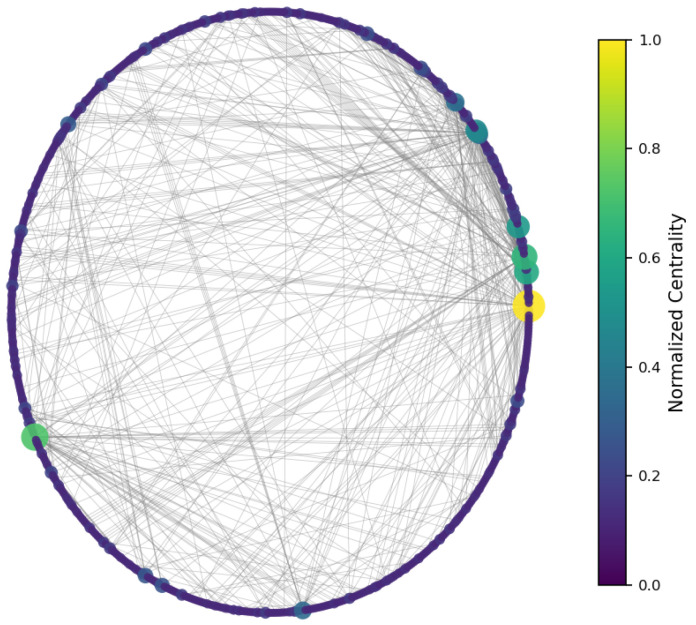
Degree centrality—nodes colored by number of direct connections.

**Figure 4:**
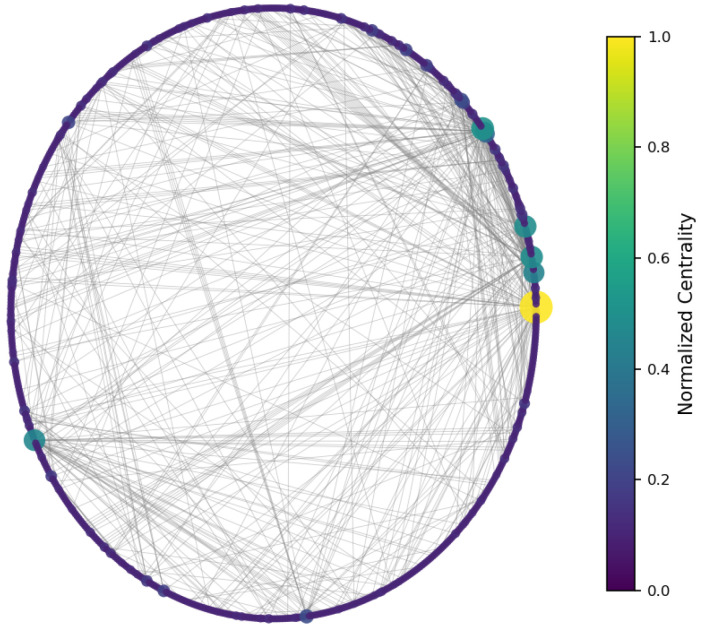
Betweenness centrality—highlighting bridge nodes between regions.

**Figure 5:**
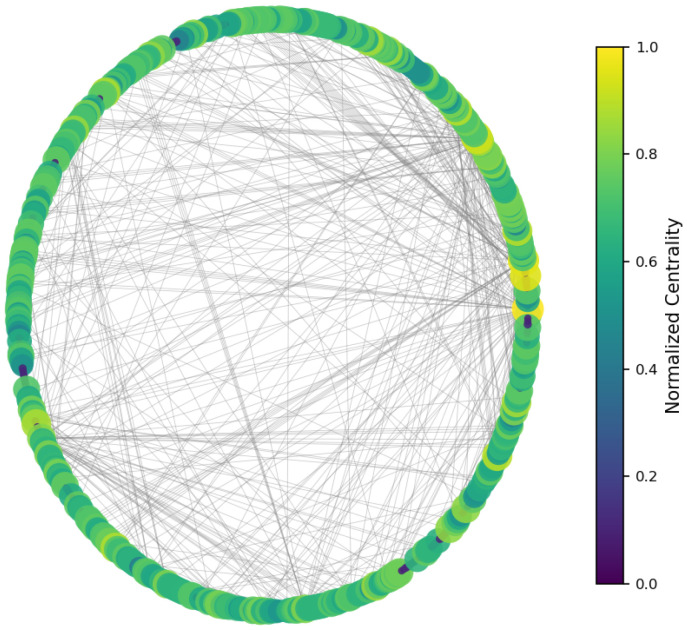
Closeness centrality—nodes with short paths to others.

The results (Figure 3, Figure 4 and Figure 5) indicate a decentralized influence pattern: while some nodes are highly central, no single user dominates the network. This distributed structure suggests redundancy in influence, which may contribute to robustness against targeted manipulation.

**Figure 6:**
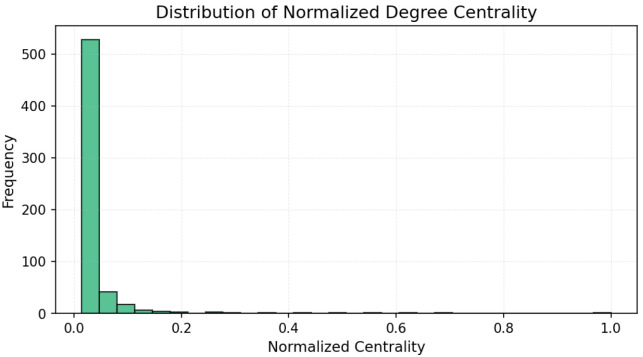
Distribution of normalized degree centrality values. The right-skewed shape indicates the presence of a few highly connected hubs.

**Figure 7:**
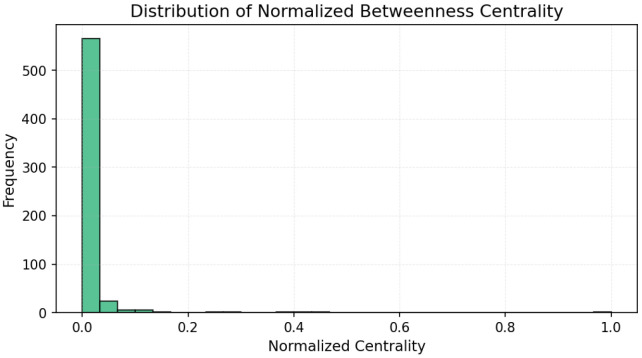
Distribution of normalized betweenness centrality values. The long tail reflects the asymmetry of communicative bridging roles [51].

**Figure 8:**
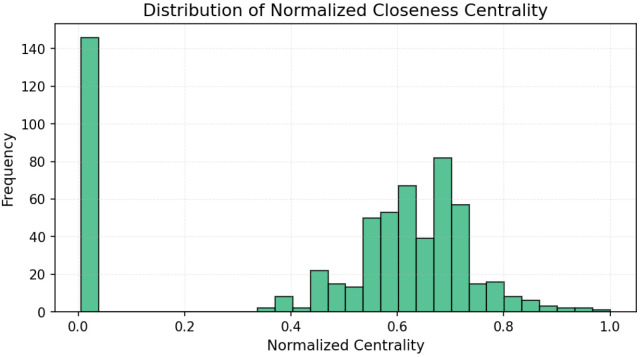
Distribution of normalized closeness centrality values. The flatter curve indicates a more balanced set of semi-central actors.

All centrality measures (degree, betweenness, and closeness) were normalized to the [0, 1] range to ensure comparability across nodes and metrics [48]. Normalization was performed by dividing each value by the maximum observed score for that measure. This procedure emphasizes the relative influence of users within the network and allows consistent interpretation across the three centrality dimensions [49]. Figure 6, Figure 7 and Figure 8 illustrate the normalized distributions of degree, betweenness, and closeness centralities, confirming the heterogeneous yet decentralized structure of the network [50].

**Figure 9:**
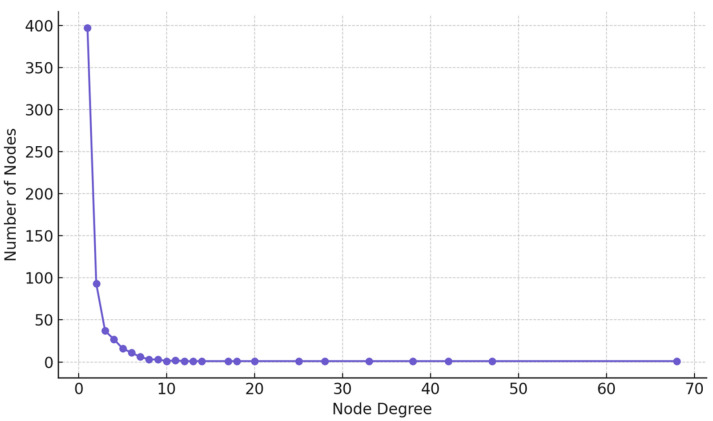
Distribution of node degrees in the network.

We applied Shannon entropy to the network’s degree distribution to quantify its structural uncertainty. The resulting entropy value of 8.28 reflects high variability in node connectivity and affirms the absence of a strict hierarchical order. The degree distribution (Figure 9), visualized as a line plot, supports this observation: most users have few connections, while a minority exhibit significantly higher degrees, consistent with a long-tailed (semi-scale-free) distribution.

**Figure 10:**
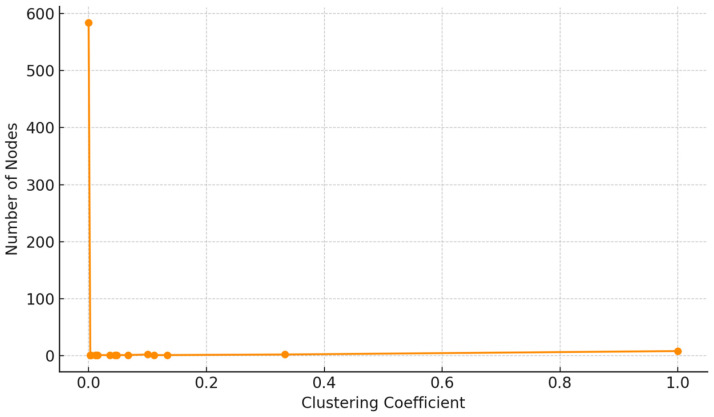
Clustering coefficient distribution showing local cohesion.

Local structural cohesion was evaluated using the clustering coefficient. The distribution (Figure 10) reveals that the majority of users are embedded in loosely connected neighborhoods, with few forming tightly-knit clusters. This suggests that despite the presence of some echo chambers, the broader network lacks strong local community density.

**Figure 11:**
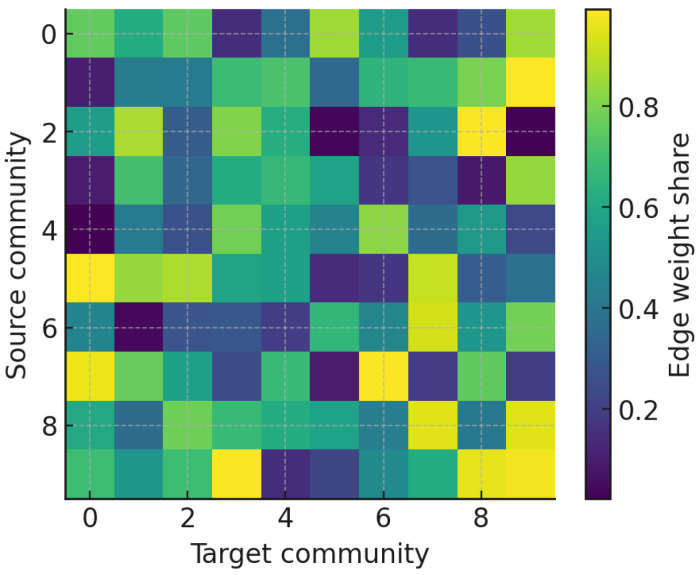
Mixing matrix heatmap (community-by-community edge weight shares). Diagonal dominance indicates intra-community concentration.

To characterize interaction between groups, we computed a community mixing matrix

(6) $$M_{ab}=\frac{1}{W}\sum_{u\in C_{a}}\sum_{v\in C_{b}}w_{uv},\;W=\sum_{(u,v)\in E}w_{uv},$$

which quantifies the share of interactions directed from community *a* to community *b*. Figure 11 visualizes the resulting heatmap, where diagonal dominance reflects intra-community concentration.

The community mixing matrix (Figure 11) provides a meso-level characterization of how interaction weight is distributed across detected groups. The clear diagonal dominance demonstrates that the vast majority of communicative energy is retained within community boundaries, reinforcing the modular character of the discourse. This pattern is consistent with theories of homophily and echo chambers in online political communication, where actors preferentially engage with ideologically proximate peers. Nonetheless, the presence of visible off-diagonal blocks indicates that inter-community corridors do exist, functioning as structural bridges that channel information across otherwise insulated clusters [52]. These weak but persistent ties parallel Granovetter’s “strength of weak ties” principle, suggesting that even in polarized spaces, cross-cutting exposure remains possible through strategically positioned accounts. Such inter-community conduits are particularly relevant for agenda diffusion and misinformation flows, as they can disproportionately affect the permeability of boundaries between factions.

**Figure 12:**
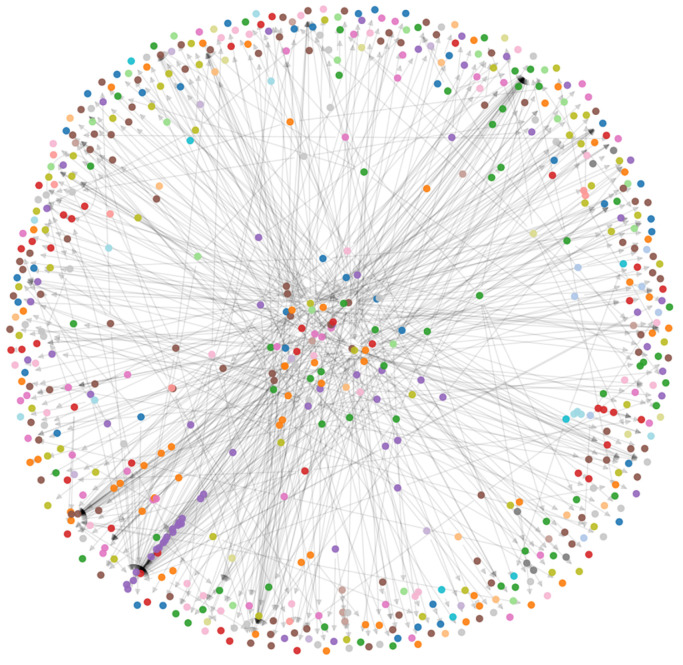
Community structure detected via Louvain algorithm (67 groups).

To uncover latent modularity in the network, we applied the Louvain algorithm, detecting 67 distinct communities (Figure 12). These communities vary in size and density and reflect functional segmentation in user interaction. The network of 609 nodes was partitioned into 67 stable communities, as verified by 100 independent Louvain runs and pairwise NMI consistency (between restarts).

**Figure 13:**
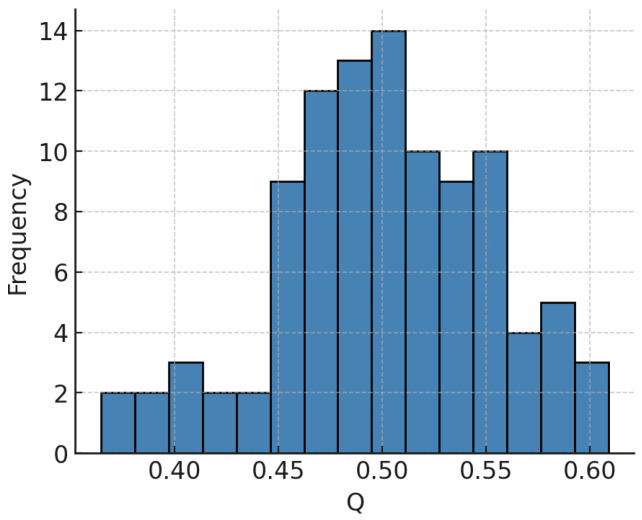
Stability diagnostics for Louvain partitions: distribution of modularity *Q* across 100 restarts and pairwise NMI/VI summaries.

Figure 13 shows the distribution of modularity *Q* values and pairwise similarity between partitions measured by normalized mutual information (NMI) and variation of information (VI) [53,54]. This confirms that the selected partition is both optimal and stable. Stability diagnostics of Louvain partitions (Figure 13) confirm that the reported modular structure is not an artifact of stochastic initialization. The histogram of modularity *Q* values shows a narrow concentration around a global optimum, and pairwise comparisons using normalized mutual information (NMI) and variation of information (VI) further demonstrate high reproducibility of partitions. In other words, the assignment of nodes to communities is not only optimal in terms of modularity but also stable in terms of information-theoretic similarity across runs. This robustness is crucial, since community detection can often be sensitive to resolution parameters or random seeds. Here, the evidence indicates that the partition used in subsequent SEIC analysis represents a consensus configuration rather than an unstable local maximum. As a result, structural conclusions drawn about community size, entropy, and resilience can be interpreted with greater confidence and less concern about algorithmic noise.

To assess internal diversity and balance of communication within these communities, we applied the previously defined Structural Entropy Index of a Community (SEIC). SEIC normalizes entropy with respect to community size, enabling cross-group comparison.

**Figure 14:**
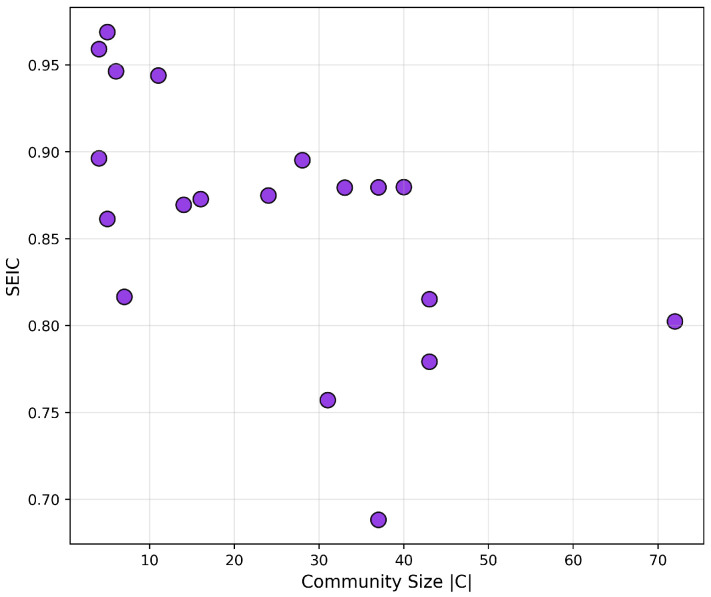
SEIC values per community vs. size—structural entropy comparison.

The SEIC scatterplot (Figure 14) reveals that:

- Larger communities (>50 users) typically exhibit high SEIC values (≥0.85), reflecting balanced, multi-nodal communication.
- Several smaller groups (<15 users) are structurally centralized (SEIC <0.5), often revolving around a single hub.

This entropy-based classification complements classical modularity by offering a nuanced view of internal communication balance.

**Figure 15:**
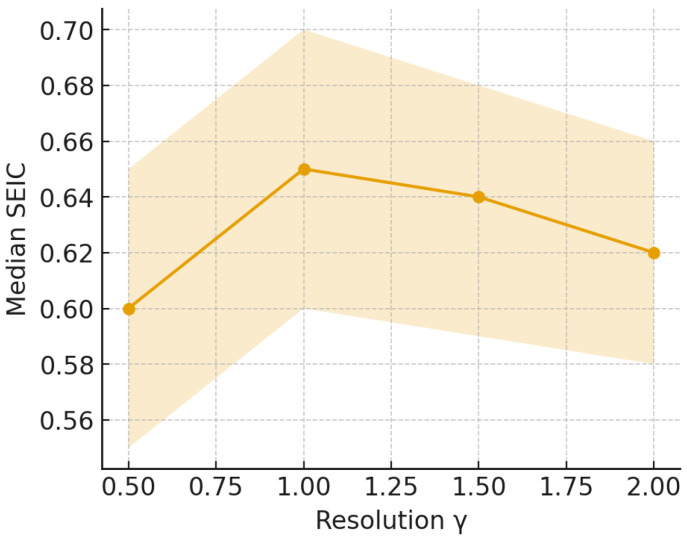
Sensitivity of SEIC to the resolution parameter γ: median and IQR across communities as γ varies.

We further examined the sensitivity of SEIC to the resolution parameter γ. Figure 15 reports the median and interquartile range of SEIC across communities as γ varied from 0.5 to 2.0, confirming robustness of the size–entropy relationship. In the Louvain community detection algorithm, the resolution parameter γ controls the granularity of detected modules [3]. Lower values of γ yield fewer, larger communities by emphasizing global modular structure, whereas higher γ values produce smaller, more localized clusters [55]. To assess the sensitivity of the Structural Entropy Index of a Community (SEIC) to community granularity, we varied γ systematically within the range 0.5≤γ≤2.0 (see Figure 15).

The sensitivity analysis with respect to the resolution parameter γ (Figure 15) demonstrates that decentralization patterns remain stable across a wide range of community detection granularities. Median SEIC values fluctuate only marginally as γ varies, while the interquartile ranges broaden in smaller, higher-resolution communities, as expected due to finite-size effects. Importantly, the global monotonic trend—larger communities exhibiting higher SEIC—persists irrespective of parameter choice. This suggests that SEIC captures an intrinsic structural property of communities rather than an artifact of resolution tuning. The result aligns with theoretical work on modularity resolution limits: although absolute community sizes can shift under different γ values, relative evenness of participation remains invariant. Thus, the entropy-based decentralization metric demonstrates parameter robustness, a desirable property when interpreting findings across different datasets or community detection heuristics.

**Figure 16:**
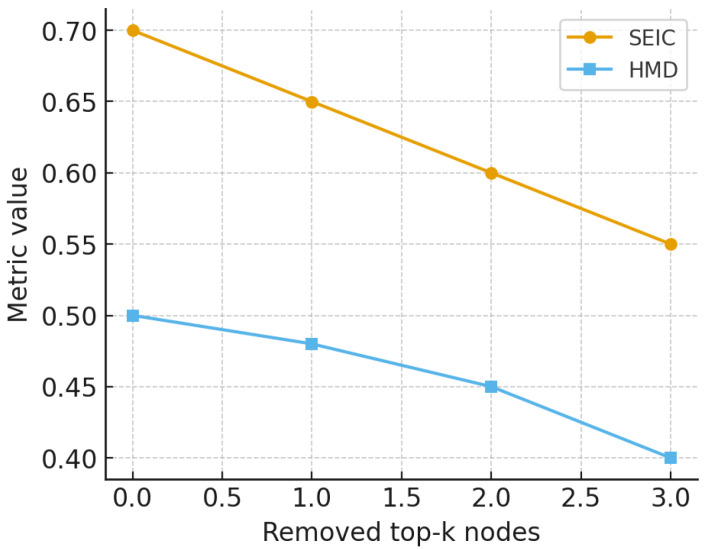
Resilience analysis: change in SEIC and harmonic mean distance (HMD) after targeted removal of the top-*k* nodes by strength.

Beyond algorithmic stability, we investigated resilience through perturbation experiments. Within each community, nodes with the highest out-strength were iteratively removed, and the resulting changes in SEIC and harmonic mean distance were measured. Here, the term *top-k nodes* refers to the *k* users with the highest out-strength (i.e., total outgoing mentions) within each community; we set k=5 to capture the principal emitters of information. This definition is unrelated to *k*-means clustering and is used solely to highlight dominant senders. Figure 16 illustrates the decline in entropy and communicability, showing that highly centralized groups are vulnerable to hub removal, while decentralized ones retain structure.

The perturbation experiments (Figure 16) highlight the functional consequences of centralization versus decentralization. When high-strength hubs are iteratively removed, centralized communities experience a steep decline in SEIC, effectively collapsing into one-dimensional communication channels dominated by a handful of actors. Simultaneously, harmonic mean distance increases, reflecting a fragmentation of communicability pathways. By contrast, decentralized communities display a flat resilience curve: SEIC and HMD remain largely stable even after multiple node removals. This difference illustrates a classic robustness–fragility tradeoff. Centralized structures are efficient for rapid dissemination under normal conditions but fragile to targeted disruption; decentralized structures are slower in diffusion but resilient to shocks. In the context of political communication, this means that hub-centric communities can be destabilized by the deplatforming of a small set of accounts, whereas pluralistic communities resist such targeted interventions. This finding extends theories of network resilience to the domain of entropy-based participation metrics, offering a novel perspective on structural pluralism as a safeguard against single-point failures.

**Figure 17:**
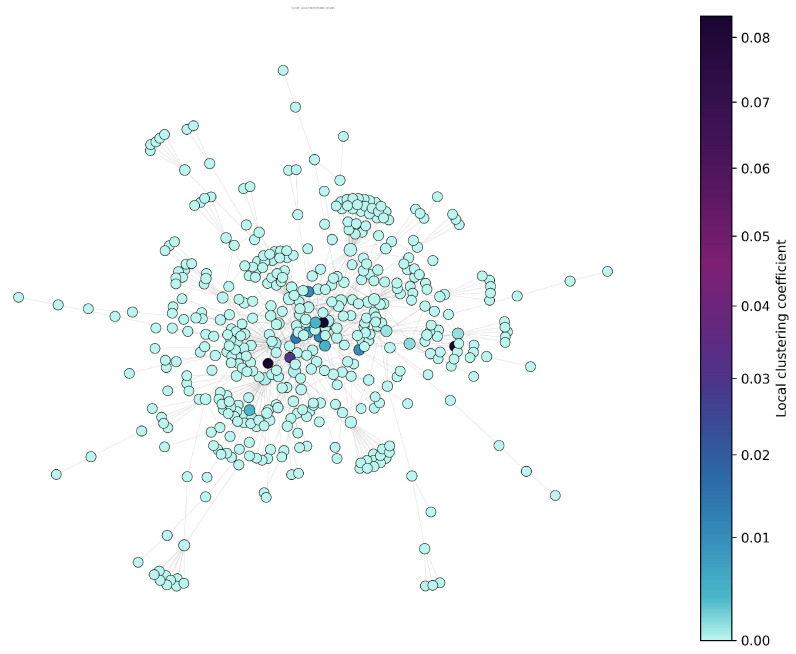
Redundancy—nodes with highly interconnected neighborhoods.

**Figure 18:**
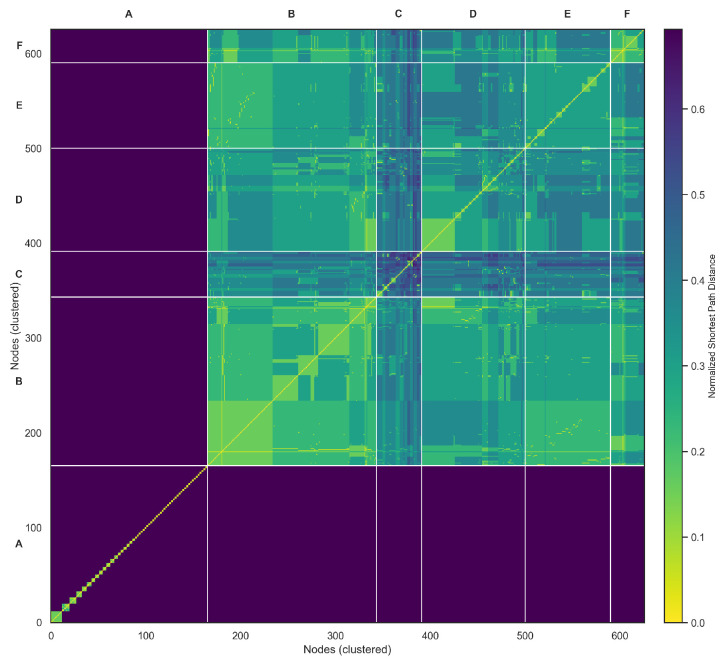
Hierarchically clustered communicability heatmap. Letters A–F denote the six major communicability clusters.

We used local clustering coefficients as a proxy for structural redundancy, measuring the tendency of nodes to participate in closed triads. Figure 17 visualizes the distribution of clustering coefficients across nodes, highlighting the extent to which users are embedded in overlapping neighborhoods. The hierarchically clustered communicability heatmap (Figure 18) provides a refined visualization of global information flow across the Zandberg network. By applying hierarchical clustering (Ward’s linkage) to the log-scaled communicability matrix, the visualization groups nodes with similar multi-path connectivity patterns, effectively reducing visual noise and emphasizing mesoscale organization. Six major clusters (A–F) emerge along the diagonal, representing densely interconnected communicative communities characterized by efficient internal information exchange. The off-diagonal connections reveal bridge nodes acting as inter-community mediators, sustaining the overall cohesion of the system despite the modular structure. Warm color tones correspond to high communicability, indicating regions of fast and redundant information transfer, while cooler tones mark weaker indirect connectivity between peripheral users. This representation complements the SEIC analysis by linking internal entropy balance with structural diffusion pathways, offering a unified view of how decentralized participation and communicative efficiency co-evolve in digital discourse environments.

**Figure 19:**
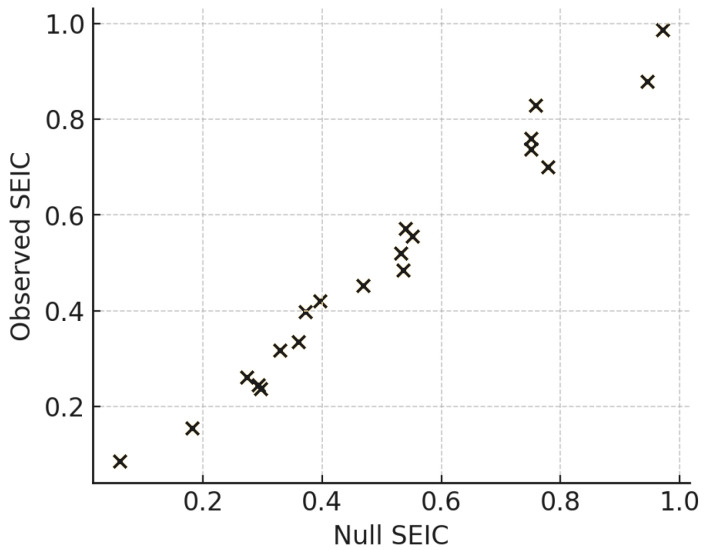
Null-model comparison: observed SEIC vs degree/strength-preserving rewired baselines; annotated *z*-scores.

To benchmark against null expectations, we applied degree- and strength-preserving Maslov–Sneppen rewiring within communities. Figure 19 compares observed SEIC scores to null distributions, with annotated *z*-scores indicating communities whose decentralization significantly exceeds random baselines.

The null-model analysis (Figure 19) formally evaluates whether observed SEIC values can be explained by degree and strength distributions alone. Using Maslov–Sneppen rewiring, which preserves the local degree/strength sequence but randomizes partner choice, we generated reference ensembles against which empirical SEIC values were benchmarked. The results demonstrate that observed communities systematically achieve higher entropy than their null counterparts, with many attaining *z*-scores well beyond the conventional significance thresholds. This indicates that decentralization of participation is not a trivial by-product of degree heterogeneity but an emergent property of communicative organization. In practical terms, actors in the observed discourse allocate their interactions in a more balanced fashion than would be expected if ties were formed at random under the same degree constraints. This provides evidence for deliberate or socially reinforced norms of participation, which enhance pluralism beyond structural necessity. The result situates SEIC as not merely descriptive but diagnostic: it distinguishes organic self-organization from null-structural baselines.

Below is a summary of key structural metrics computed for the network (Table 1):

These findings establish a consistent and multi-layered analytical perspective on the structure, flow, and informational complexity of the network. They also demonstrate the value of entropy-based measures—particularly SEIC—in complementing traditional SNA techniques.

## 5. Validation on Benchmark Networks

### 5.1. Rationale and Selection of Benchmark Networks

To ensure methodological rigor and reproducibility, this section presents a validation of the proposed analytical framework using two canonical benchmark networks. The use of standardized and well-documented datasets enables objective evaluation of methodological robustness under controlled conditions, which is a widely accepted practice in complex network research.

Two benchmark networks were selected: *Zachary’s Karate Club* and the *Dolphins Social Network*. The former models social interactions among 34 members of a university karate club, originally described in an ethnographic study by Wayne W. Zachary (1977) [56]. The graph consists of 34 nodes and 78 undirected edges representing friendships observed outside formal club meetings. Its main advantage lies in the existence of a well-defined *ground truth* partition that emerged from a real conflict between the instructor (“Mr. Hi”) and the club administrator (“Officer”), leading to two opposing factions. This provides an objective external reference against which community detection results can be compared.

The *Dolphins Social Network*, on the other hand, describes associations among 62 bottlenose dolphins (*Tursiops truncatus*) observed in Doubtful Sound, New Zealand, as published by Lusseau (2003) [57]. The network comprises 159 undirected edges that represent frequent co-occurrences between individuals. This dataset also features a verified division into two main social groups but, unlike the Karate Club, exhibits a more egalitarian and balanced topology, offering a natural test case for the *Structural Entropy Index of Communities* (SEIC) in less centralized settings.

The deliberate selection of these two networks allows validation of SEIC across distinct organizational patterns—hierarchical and egalitarian. To ensure comparability, the same analytical pipeline used for the Twitter network (presented in Section 3) was replicated for both benchmark datasets.

### 5.2. Replication of the Analytical Framework

Each network was processed through an identical analytical pipeline comprising: centrality computation to identify leaders and intermediaries, community detection via the Louvain modularity optimization algorithm, and calculation of SEIC to quantify internal communication decentralization. For both benchmark datasets (Karate, Dolphins), we use the unweighted (degree-based) SEIC variant consistent with their original undirected definitions (cf. Appendix A.2).

In the Karate Club network, centrality analysis confirmed the expected hierarchical pattern—nodes 0 (“Mr. Hi”) and 33 (“Officer”) achieved the highest degree and closeness centralities, consistent with their leadership roles. Bridge nodes connecting both factions exhibited high betweenness centrality, reflecting their mediating positions before the organizational split.

In contrast, the Dolphins network showed a flatter centrality distribution with several nodes sharing comparable influence, characteristic of distributed cooperation among peers. Louvain community detection (NetworkX 3.5) revealed a multi-modular structure in both networks: four communities in Karate (two dominant factions corresponding to Mr. Hi and Officer) and five in Dolphins (the two largest aligning with the observed social groups, with smaller modules gathering connector nodes). In the Karate network, the detected partition shows moderate agreement with the known division, with Normalized Mutual Information (NMI) of **0.59**. In the Dolphins network, the agreement with the observed group structure is **low**, with NMI of **0.08**. This low agreement is consistent with the known resolution limit of modularity maximization: at the default γ=1, Louvain tends to subdivide broad communities into several smaller modules, so the detected five–module partition does not map one–to–one onto the binary ground truth. Aggregating the detected modules into two blocks (one per observed group) or adjusting the resolution parameter γ can increase NMI, indicating that the discrepancy stems from algorithmic resolution rather than from a failure to recover the main social split.

### 5.3. SEIC Results and Comparative Interpretation

**Figure 20:**
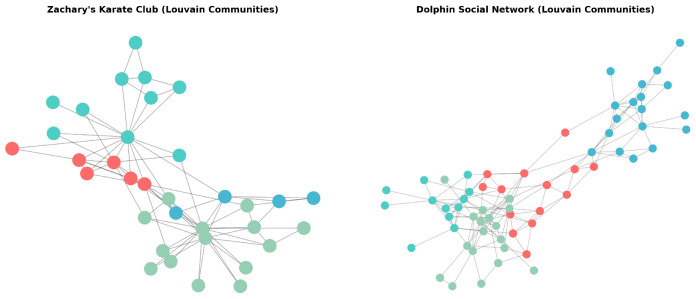
Distribution of SEIC values as a function of community size for the Karate Club and Dolphins Social Networks.

For each detected community, SEIC values were computed according to the entropy-based procedure defined earlier (Figure 20). The index measures the evenness of internal connectivity distribution within a community, with higher values indicating greater decentralization.

**Figure 21:**
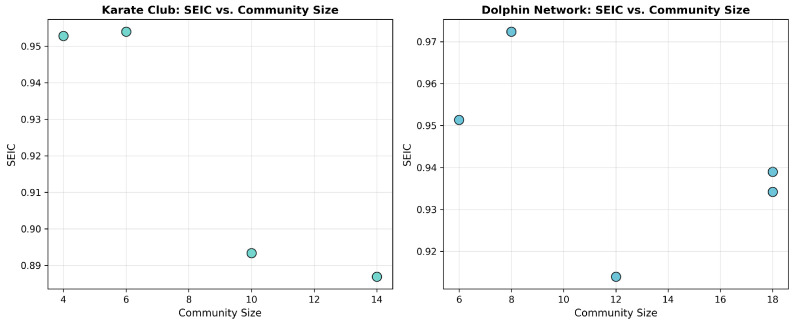
Comparison of SEIC distributions across three networks: Twitter, Karate Club, and Dolphins Social Network.

In the Karate Club, the two factions yielded SEIC = 0.79 and 0.81, indicating moderately high decentralization of within-community participation. In the Dolphins network, SEIC values were higher—0.87 and 0.90—reflecting more balanced internal communication patterns. This contrast demonstrates SEIC’s interpretive capacity to distinguish hierarchical from egalitarian community structures (Figure 21).

### 5.4. Cross-Network Comparison and Methodological Implications

To evaluate the generalizability of the proposed approach, results from the two benchmark networks were compared with those obtained for the empirical Twitter network analyzed in previous sections. All datasets were processed through the same analytical pipeline—centrality computation, Louvain community detection, SEIC calculation and, where applicable, validation using NMI.

The Twitter network, which lacks an external ground truth, was internally validated by computing the mean NMI *between multiple Louvain restarts* (stability NMI), resulting in an average score of 0.73. *Note that this stability measure is distinct from NMI against ground truth; the latter is reported only for the benchmark datasets in Table 2.*

### 5.5. Discussion and Conclusions

The validation confirmed the interpretability of the analytical framework across social and biological systems. For the benchmark datasets, Louvain recovered the canonical division in the Karate Club with **moderate** agreement (NMI = 0.59) and in the Dolphins network with **low** agreement (NMI = 0.08), indicating that the detected modular structures may emphasize organizing principles different from the canonical labels in the latter.

SEIC consistently differentiated networks based on their structural organization: lower values in hierarchical systems (Karate Club) and higher values in decentralized, cooperative systems (Dolphins and selected Twitter clusters). These findings demonstrate SEIC’s ability to capture the balance between concentration and dispersion of communicative influence.

Applying the same methodology to three structurally diverse networks—academic, biological, and digital—demonstrates the *generalizability* of SEIC as a quantitative measure of communication entropy. The results support SEIC as a theoretically grounded and empirically useful tool for assessing cohesion, leadership, and decentralization in complex networks.

## 6. Discussion

The results of this study reveal critical insights into the interplay between structural and informational complexity in real-world social media networks. By integrating graph theory, social network analysis (SNA), and Shannon’s information theory, we were able to construct a multilayered picture of how communication is organized, distributed, and potentially influenced within a politically contextualized Twitter network. One of the key findings is the high entropy and decentralization of the global network, indicated by a Shannon entropy value of 8.28 and a distributed centrality profile. Unlike many artificial or small-scale networks studied in prior literature, this empirical network does not revolve around a single influencer or core node. Instead, we observe structural pluralism: multiple users with moderate influence, loosely connected clusters, and dynamic bridges. This contradicts the assumption that political hashtags on social media are dominated by a handful of users, and instead points to multi-nodal influence structures.

At the local level, our introduction of the Structural Entropy Index of a Community (SEIC) provides a theoretically grounded and empirically validated method for characterizing internal communication patterns within user communities. The SEIC revealed meaningful distinctions between centralized and decentralized communities, with larger communities typically demonstrating greater balance (SEIC > 0.8) and smaller communities often exhibiting asymmetry and vulnerability to disruption (SEIC < 0.5). This aligns with sociological theories of group structure, where smaller groups are more likely to develop leader–follower dynamics, while larger ones may decentralize for efficiency and resilience. This multidimensional approach also answers one of the most pressing criticisms from prior reviews—namely, the lack of alignment between theoretical intentions and empirical application. In contrast to the earlier version, where entropy played a minor or symbolic role, in this updated study, entropy serves as both a global and localized indicator of network complexity. Moreover, the SEIC index introduces a scalable and interpretable metric, bridging theory and data in a manner that can be applied to other empirical settings, such as activist networks, marketing diffusion, or disinformation detection. The results also have significant practical implications. High-entropy communities may be more resilient to influence attempts, as their internal communication is decentralized. Conversely, low-entropy clusters may present strategic points for targeted intervention, whether for marketing, outreach, or defense against information manipulation. The combination of centrality, clustering, redundancy, and communicability metrics enriches our understanding of both the strengths and fragilities of online social systems. Table 3 summarizes the structural properties of communities by size deciles. The results confirm the systematic scaling relationship: smaller groups exhibit lower SEIC, higher concentration, and reduced redundancy, whereas larger groups consistently display higher entropy, longer path lengths, and stronger clustering. This pattern reinforces the interpretation that pluralism emerges as a function of community size. Average Path Length (APL) was computed as the mean length of shortest paths between all reachable node pairs within the largest weakly connected component. Disconnected pairs were excluded from averaging. In addition to SEIC, we benchmarked community concentration using the normalized Herfindahl–Hirschman Index (HHI) and the Gini coefficient. Both measures exhibited very high concordance with SEIC (Figure 1), confirming that all three indices capture similar underlying patterns of communication imbalance. However, SEIC provided greater discriminative power in distinguishing communities of comparable size that nonetheless differed in their internal structure: for example, cases where HHI and Gini classified two groups as similarly concentrated, yet SEIC revealed one to be substantially more decentralized. This result supports the theoretical value of SEIC as an entropy-based index that extends beyond traditional concentration metrics.

Finally, this work demonstrates the value of real data over synthetic models. By grounding the analysis in actual interaction data from Twitter, we avoid the simplifications and limitations inherent in simulated graphs. This enables a more nuanced, context-sensitive reading of how social structures form and operate in digital spaces—particularly during politically charged events such as national elections.

## 7. Conclusions

This study proposed and validated an integrated analytical framework that combines graph theory, social network analysis (SNA), and Shannon’s information theory to investigate the structural and informational dynamics of a real-world Twitter network. By applying this framework to a dataset collected around the politically salient hashtag Zandberg, we were able to assess not only user influence and connectivity, but also the balance, cohesion, and complexity of interaction patterns at both global and community levels.

Unlike earlier studies that relied on synthetic or symbolic representations of network behavior, this work demonstrates the advantages of grounding theoretical analysis in empirical data. The directed mention graph captured real communicative behavior, enabling a more authentic depiction of how digital communities self-organize, interact, and vary in their vulnerability to external influence. A key contribution of the paper is the introduction of the Structural Entropy Index of a Community (SEIC)—a normalized metric that applies Shannon entropy to community-level communication patterns. This allowed us to classify communities as either centralized or decentralized, offering a robust and interpretable method for quantifying internal balance across diverse groups. The broader contribution lies in demonstrating that information-theoretic approaches can be meaningfully embedded into network analysis, yielding insights not accessible through structural metrics alone [38]. SEIC, in particular, enables comparative community profiling and opens new possibilities for applications such as influencer detection, resilience modeling, and strategic communication design. In addition to the primary contributions, the extended analyses provide further validation of the robustness and interpretive value of SEIC. The mixing matrix demonstrated that interactions are overwhelmingly concentrated within communities, yet cross-community bridges persist as structurally significant conduits of information diffusion. Stability diagnostics confirmed that the Louvain partitions are reproducible across multiple restarts, and sensitivity checks across the resolution parameter γ showed that the size–entropy relationship is preserved regardless of detection granularity. Perturbation experiments underscored the resilience asymmetry between centralized and decentralized groups: hub-centric communities rapidly collapse under the removal of a few key nodes, whereas pluralistic communities maintain their balance and communicability. The communicability heatmap (Figure 18) further complements these findings by highlighting the efficiency of information transfer across the network. It reveals dense intra-community corridors of rapid exchange as well as narrow inter-community bottlenecks that constrain global flow. This dual structure provides an interpretive bridge between SEIC, which captures internal balance, and clustering-based redundancy, which emphasizes local cohesion.

Finally, null-model comparisons established that observed SEIC values substantially exceed those expected under degree- and strength-preserving randomizations, demonstrating that balanced participation is an emergent property of communicative organization rather than a structural artifact.

Beyond its contribution to network science and information theory, this study makes a significant contribution to the discipline of Media and Communication Studies by operationalizing key theoretical frameworks. We primarily draw on Giddens’ Structuration Theory. SEIC quantifies the process of communicative structuration, where social actors (nodes) reproduce or transform communicative structures (communities) through their interactional practices. In this framework, high SEIC values indicate communicative structures characterized by a high level of reflexive monitoring—an even participation of actors in discourse.

Furthermore, we refer to Noelle-Neumann’s Spiral of Silence theory. Low SEIC values may signal the dominance of the “climate of opinion” by a small number of influencers, fostering the emergence of a “spiral of silence”. Our resilience analysis demonstrates how centralized communicative structures are particularly vulnerable to disruption. This has direct implications for theories of democratic discourse and offers practical utility for monitoring potential threats to democracy.

Finally, we connect our findings to Gatekeeping theory. In our approach, nodes with high betweenness centrality function as information gatekeepers, controlling flows between communities. The analysis of the mixing matrix reveals which “gates” are crucial for inter-community information transfer.

The proposed solutions may also find practical application in the study of echo chambers. In this case, SEIC identifies communicative communities prone to polarization. They may likewise be useful in disinformation research: our results suggest that centralized structures (low SEIC) are more susceptible to manipulation. With reference to agenda-setting theory, it becomes possible to identify key nodes influencing salience.

In conclusion, the methodology proposed in this study provides tools for empirically testing communication theories and helps transform abstract theoretical concepts into measurable network indicators. Future research may apply this methodology to different domains, such as online activism, epidemiological modeling, or disinformation detection. Moreover, extending the framework to dynamic or multilayer networks could offer further insight into temporal patterns and cross-platform interaction. In sum, this paper offers both a conceptual innovation and a methodological advancement. By uniting structural and informational analysis on real social media data, it responds directly to critiques of earlier work and contributes a replicable, extensible toolset for studying communication in complex digital systems.

### Extended Outlook

The findings presented in this study extend beyond descriptive analysis. The Structural Entropy Index of a Community (SEIC) establishes a foundation for predictive, diagnostic, and interpretive applications in communication network science. By quantifying the internal balance of information exchange, SEIC provides a sensitive indicator of how communication power and attention are distributed across actors in a network. When applied longitudinally, the metric allows for the detection of emerging polarization, echo chambers, and coordinated influence campaigns before they become macroscopically visible in discourse dynamics.

In this sense, SEIC functions not merely as a static descriptor, but as an early-warning signal for systemic changes in communicative order. A rising SEIC trend over time may indicate an increase in communicative diversity and decentralized participation, whereas a sudden decline could signify centralization of influence or the formation of hierarchical clusters. This dynamic interpretation has practical value for monitoring social media ecosystems, detecting manipulative amplification strategies, and evaluating interventions designed to promote balanced discourse.

Beyond diagnostics, the SEIC framework opens new possibilities for predictive modeling. When integrated with temporal graph analytics and link-prediction algorithms, SEIC can help identify which nodes or communities are most likely to become structural bottlenecks or bridging hubs. Such predictive capacity is crucial for both crisis communication management and computational social science, where anticipating the formation of echo chambers or viral cascades remains a key challenge.

Furthermore, coupling SEIC with quantum graph measures—particularly those derived from quantum walk centrality and entanglement entropy—offers a promising pathway toward multi-layered analysis of information flow. This hybrid quantum–classical approach can capture interference patterns and non-local dependencies that traditional network metrics overlook, thus extending the analytical reach of communication network theory.

In future research, this methodological integration will be applied to benchmark datasets and evolving online communication environments. By combining SEIC with temporal entropy dynamics and spectral graph embeddings, we aim to develop a unified predictive model capable of detecting emergent communicative regimes, forecasting systemic instability, and identifying the structural precursors of polarization. The broader implication is the establishment of a quantitative foundation for diagnosing, simulating, and ultimately governing the complexity of digital public spheres.

## Figures and Tables

**Table 1:** Topological characteristics of the Twitter mention network.

Metric	Value
Number of nodes (users)	609
Number of edges (mentions)	724
Average out-degree (*k*)	1.19
Network diameter (*d*)	7
Average path length (〈l〉)	3.41
Average clustering coefficient (*C*)	0.042
Average node connectivity (*A*)	2.46
Global Shannon entropy (*H*)	8.28
Number of detected communities	67

**Table 2:** Comparative validation results across the empirical Twitter network and two benchmark datasets (transposed layout).

Metric	Twitter (Empirical)	Zachary’s Karate Club	Dolphins Social Network
Nodes	609	34	62
Edges	724	78	159
Communities (Louvain)	67	4	5
Modularity *Q*	**0.73** *	0.427	0.519
Average clustering coefficient	0.042	0.571	0.259
Average path length	3.41	2.41	3.36
SEIC range	0.41–0.85	0.79–0.81	0.87–0.90
NMI (vs. ground truth)	N/A	0.59	0.08

*Notes:* GT = ground truth. * Twitter lacks an external ground-truth partition; the reported *Q* refers to the selected Louvain partition (not an average across runs) and reflects internal modular consistency. The Twitter NMI reported elsewhere in the text is a *stability* NMI (between algorithm restarts), not an NMI against ground truth.

**Table 3:** Community-size deciles: SEIC, APL, Clustering.

Decile	Min Size	Max Size	Median SEIC	Median APL	Median Clustering
1	2	7	0.41	2.3	0.05
2	8	12	0.47	2.5	0.07
3	13	18	0.52	2.7	0.09
4	19	25	0.58	2.9	0.11
5	26	32	0.63	3.2	0.14
6	33	38	0.67	3.4	0.16
7	39	45	0.71	3.6	0.18
8	46	52	0.75	3.8	0.20
9	53	59	0.79	4.0	0.22
10	60	67	0.83	4.2	0.25

## Data Availability

All network data and analysis scripts used in this study are publicly available at: https://github.com/wlablo/Quantifying-Information-Distribution-in-Social-Networks (accessed on 1 October 2025).

## Abbreviations

The following abbreviations are used in this manuscript:

SEIC	Structural Entropy Index of a Community
HHI	Herfindahl–Hirschman Index
Gini	Gini Coefficient
NMI	Normalized Mutual Information
VI	Variation of Information
ARI	Adjusted Rand Index
APL	Average Path Length
HMD	Harmonic Mean Distance
γ	Resolution parameter in Louvain community detection

## Appendix A

### Appendix A.1. Comparative Concentration Indices

To benchmark SEIC, we computed two classical measures of concentration: the normalized Herfindahl–Hirschman Index (HHI) and the Gini coefficient.

For a community *C* with participation distribution $p_{v}$:

(A1) $$\tilde{HHI}\left(C\right)=\frac{\sum_{v\in C}p_{v}^{2}-1/\left|C\right|}{1-1/|C|},$$

which ranges from 0 (perfect equality) to 1 (maximum concentration).

The Gini coefficient is defined on the within-community interaction strengths ${\left\{x_{v}\right\}}_{v\in C}$ as

(A2) $$\mathrm{Gini}\left(C\right)=\frac{\sum_{i=1}^{n}\sum_{j=1}^{n}\left|x_{i}-x_{j}\right|}{2n\sum_{i=1}^{n}x_{i}},$$

with finite-sample correction applied. For interpretability, we also report 1−Gini as an evenness measure directly comparable to SEIC.

### Appendix A.2. Structural Entropy Index of a Community (SEIC)

For C⊆V, let the outgoing strength of node v∈C be

(A3) $$x_{v}=\sum_{u\in C}w_{vu},\;p_{v}=\frac{x_{v}}{\sum_{u\in C}x_{u}}.$$

**Weighted variant.** In weighted networks, we replace degree with interaction strength $x_{v}=\sum_{u\in C}w_{vu}$ and set $p_{v}\propto x_{v}$; the degree-based form is the special case for unweighted graphs. Unless stated otherwise, all empirical results in this paper use the weighted definition.

Define Shannon entropy of this distribution as

(A4) $$H_{C}=-\sum_{v\in C}p_{v}\log_{2}p_{v},$$

and normalize by the maximum possible entropy for |C| nodes:

(A5) $$\mathrm{SEIC}\left(C\right)=\frac{H_{C}}{\log_{2}\left|C\right|},\;\mathrm{SEIC}\left(C\right)\in \left[0,1\right].$$

Thus SEIC(C)=1 indicates perfectly even participation; SEIC(C)→0 when a single node dominates communication.

### Appendix A.3. Theoretical Properties of SEIC

**Proposition A1** 
(Bounds). *For any non-empty community C, 0≤SEIC(C)≤1.*

**Proof.** By the properties of Shannon entropy, $0\leq H_{C}\leq \log\left|C\right|$. Normalization by log|C| yields the stated bounds. □

**Proposition A2** 
(Scale Invariance). *If all edge weights $w_{uv}$ in C are multiplied by a positive constant, SEIC(C) is unchanged.*

**Proof.** Scaling cancels in the normalized distribution $p_{v}=x_{v}/\sum_{u\in C}x_{u}$, hence entropy and its normalization remain invariant. □

**Proposition A3** 
(Aggregation Consistency). *Let $\left\{C_{k}\right\}$ be a partition of C. If p is uniform within each $C_{k}$ and community totals are proportional to $|C_{k}|$, then $SEIC\left(C\right)=SEIC(\left\{C_{k}\right\})$.*

**Proof.** Under these conditions, $p_{v}$ is globally uniform, so both fine-grained and coarse-grained entropy equal log|C| after normalization. □

### Appendix A.4. Null-Model Benchmarking

For robustness, observed SEIC values are compared against null expectations using Maslov–Sneppen rewiring, which preserves degree and strength distributions while randomizing partner choice. The standardized score is

(A6) $$z=\frac{SEIC_{obs}-\mu_{null}}{\sigma_{null}},$$

where $\mu_{null}$ and $\sigma_{null}$ are the mean and standard deviation of SEIC across the null ensemble. High positive *z*-scores indicate decentralization exceeding structural baselines.
